# Longus colli calcific tendinitis, an uncommon cause of neck pain. A short series of cases and review of literature

**DOI:** 10.1007/s00264-025-06713-y

**Published:** 2025-12-18

**Authors:** Andrés Combalia, Kevin Zelada-Castro, Xavier Alemany, Caribay Vargas-Reverón, Ernesto Muñoz-Mahamud

**Affiliations:** 1 Departament de Cirurgia i Especialitats Medicoquirúrgiques, Facultat de Medicina i Ciències de la Salut, Universitat de Barcelona (UB), Barcelona, Spain, Barcelona, Spain; 2 Department Orthopedic Surgery and Trauma, Hospital Clinic of Barcelona, University of Barcelona, Barcelona, Spain; 3 Institut d’Investigacions Biomèdiques August Pi i Sunyer (IDIBAPS), c. Villarroel, 170, 08036 Barcelona, Spain

**Keywords:** Longus colli calcific tendinitis, Neck pain

## Abstract

**Purpose:**

Longus colli acute calcific tendinitis (LCCT) is a painful disease characterized by a triad of neck pain, neck stiffness and odynophagia. It is a relatively rare cause of neck pain, often unknown or underdiagnosed, but it is important to be aware of its existence as it can mimic other potentially more dangerous illnesses.

**Methods:**

We present a short series of five cases in which we gathered demographic and clinical data including imaging studies and compared our findings to previous reports by other authors. The diagnosis of LCCT was made by the combination of a compatible clinical presentation and blood workup plus the identification of a calcific deposit in the proximal oblique fibers of the longus colli muscle and retropharyngeal edema via computed tomography.

**Results:**

Five patients were analyzed. Mean age was 44 years, three female and two male. All patients initially presented neck pain and painful mobilization, while only 60% presented with odynophagia. There were no patients with radiculopathy nor fever. The mean values for ESR, CRP and White Blood Cell (WBC) were 23.2 mm/h, 2.97 mg/dl and 10.21 * 10^9/L respectively. On CT and/or MRI exploration all the patients presented a visible calcific deposit on the anteroinferior border of the anterior C1 arch and visible signs of retropharyngeal oedema.

**Conclusions:**

LCCT is a self-limited pathology that is caused by a foreign-body type reaction in the retropharyngeal space secondary to the degradation and resorption of calcium hydroxyapatite deposits usually found at the anteroinferior border of the anterior C1 arch. It is necessary to create awareness of this pathology amongst physicians because it can mimic more serious illness like retropharyngeal abscess, meningitis and spondylodiscitis and this may lead to unnecessary expenditures and antibiotic usage.

## Introduction

Acute longus colli tendinitis in an inflammatory process that usually takes place on the upper oblique fibres of the longus colli tendon and retropharyngeal space. The proposed pathogenesis is the deposition of hydroxyapatite crystals that produce a local foreign body inflammatory response which is believed to be responsible for the patients’ symptomology. There is increased interest in creating awareness on this pathology because it can be easily misdiagnosed as a retropharyngeal abscess, meningitis or spondylodiscitis, directly impacting on patient’s comfort and in unnecessary use of antibiotic therapy. The gold standard for diagnostic is the presence of a calcific deposition usually on the anteroinferior border of the anterior C1 arch, and the presence of oedema on the retropharyngeal space. There could also be a slight C-reactive protein (CRP) and Erythrocyte Sedimentation Rate (ESR) elevation along with mild pyrexia. Treatment usually consists of non-steroidal anti-inflammatory drugs, corticosteroids and in some cases opioids or cervical immobilization.

## Methods

We present 5 cases attended in the emergency department of our hospital with symptomatology consisting mainly of neck pain, neck stiffness and odynophagia. All patients were admitted into the emergency room and blood workup was done searching for inflammatory response. Evaluation by the otorhinolaryngology team was carried on when considered necessary, and a Computed Tomography (CT) scan was performed in all patients looking for characteristic findings. In some of the patients further contrast Magnetic Resonance Imaging (MRI) evaluation of the retropharyngeal edema was done to rule out the presence of an abscess and to evaluate the integrity of the cervical spine. The data of all patients was gathered and is presented on Table 1.

**Table 1:** Demographic and clinical data of patients included in this study. ESR erythrocyte sedimentation Rate; CRP C-reactive protein; WBC white blood Cell; CT computed tomography

Patient	Sex	Age	Days before consultation	Neck Pain	Radicular Pain	Painful mobilization	Odynophagia	Fever	ESR mm/h	CRP mg/dl	WBC x10^9/L	CT calcific deposition C1-C2	Retropharyngeal edema
1	M	38	1	yes	no	yes	no	no	25	1.4	8.5	yes	yes
2	F	50	6	yes	no	yes	yes	no	15	4.35	8.49	yes	yes
3	F	45	2	yes	no	yes	yes	no	26	0.87	9.88	yes	yes
4	F	49	3	yes	no	yes	no	no	30	2.62	13.3	yes	yes
5	M	38	4	yes	no	yes	yes	no	20	5.59	10.9	yes	yes

## Results

Five patients were analyzed. Mean age was 44 years, three female (60%) and 2 male (40%). All patients in the series initially presented neck pain and painful mobilization, while only 60% presented with odynophagia. There were no patients with radiculopathy nor fever. The mean values for ESR, CRP and White Blood Cell (WBC) were 23.2 mm/h, 2.97 mg/dl and 10.21 * 10^9/L respectively. On CT evaluation all of the patients presented a visible calcific deposit on the anteroinferior border of the anterior C1 arch and visible signs of retropharyngeal edema. Results are presented in Table 2.

**Table 2:** Mean data of patients (ESR erythrocyte sedimentation Rate; CRP C-reactive protein; WBC white blood Cell)

Domain	Mean Value
age	44 yr
sex	f (60%), m (40%)
days before consultation	3.2 days
neck pain (%)	100%
radiculopathy (%)	0%
painful mobilization (%)	100%
odynophagia (%)	60%
fever (%)	0%
ESR mm/H	23.2 mm/h
CRP mg/dL	2.97 mg/dl
WBC * 10^9/L	10.21 * 10^9/L
c1-c2 calcific deposit on CT	100%
retropharyngeal edema	100%

## Discussion

The first ever case report on Calcific Longus Colli Tendinitis seems to be reported by Hartley in 1964 [1–4. Since then few case reports have been made so the knowledge of this pathology is still scarce among physicians.

Longus Colli Muscle is located in the prevertebral area and consists on three segments, upper oblique fibres, vertical fibres and lower oblique fibres, spanning from the anterior arch of C1 and running bilaterally on the anterolateral border of the cervical vertebral bodies and anterior tubercles of their transverse processes down to the anterior portion of T1 - T3 [5–7], it is manly a cervical flexor, ipsilateral side flexor and it has a certain degree of cervical rotation.

Acute Longus Colli Calcific Tendinitis (LCCT) presents most commonly with the triad consisting of Neck Pain, Neck stiffness and Odynophagia, and a slight elevation of ESR and C-reactive protein [6, 8–14] although others have reported fever [15], trismus [7] and torticollis [6, 16] as accompanying symptoms, although none of the patients presented in this small series had fever.

LCCT has often a self-limiting course that is resolved in one to three weeks in patients from 30 to 60 years [6, 10, 17], and it is believed that it’s caused by the deposition of hydroxyapatite crystals on the upper oblique fibers of the Longus Colli Muscle (LCM), often at C1-C2 level. Rupture and resorption of these crystals cause an aseptic foreign-body type inflammatory response and results in the formation of reactive fluid in the retropharyngeal space surrounding the muscle, which is believed to be the cause of neck pain and the accompanying symptoms. Recently Yamamoto et al. [17] described three stages of the disease: pre-calcification, calcific, and post-calcific. During the resorptive stage of the calcific stage, calcific deposits are invaded by macrophages, polymorphonuclear cells and fibroblasts. This is the most painful stage and it is believed that the calcific deposits are being replaced by granulation tissue. Others have studied the possible causes that lead to calcific depositions in tendons like the supraspinatus tendon and Achilles tendon, and have proposed five stages of the disease pre-calcific, formative, resting, resorptive and post-calcific. The pre-calcific phase is characterized by fibrocartilaginous metaplasia of tendon cells that leads to the formation of calcium foci separated by fibrocartilage cells (formative phase). Posteriorly comes the resting phase where the multifocal calcium deposition presents, surrounded by the metaplastic fibrocartilage cells, that subsequently gets surrounded by macrophages/multinucleated cells that phagocyte calcific debris -this is believed to be the most painful phase-, finally resulting in the post-calcific phase where the calcific deposit gets reabsorbed and is replaced by granulation tissue [18, 19]. Rui et al. [20] suggested a possible erroneous differentiation of tendon-derived stem cells into chondrocytes or osteoblasts may be the possible mechanism leading to calcific tendinopathy, although the exact mechanism why this happens is yet to be clarified.

**Fig. 1:**
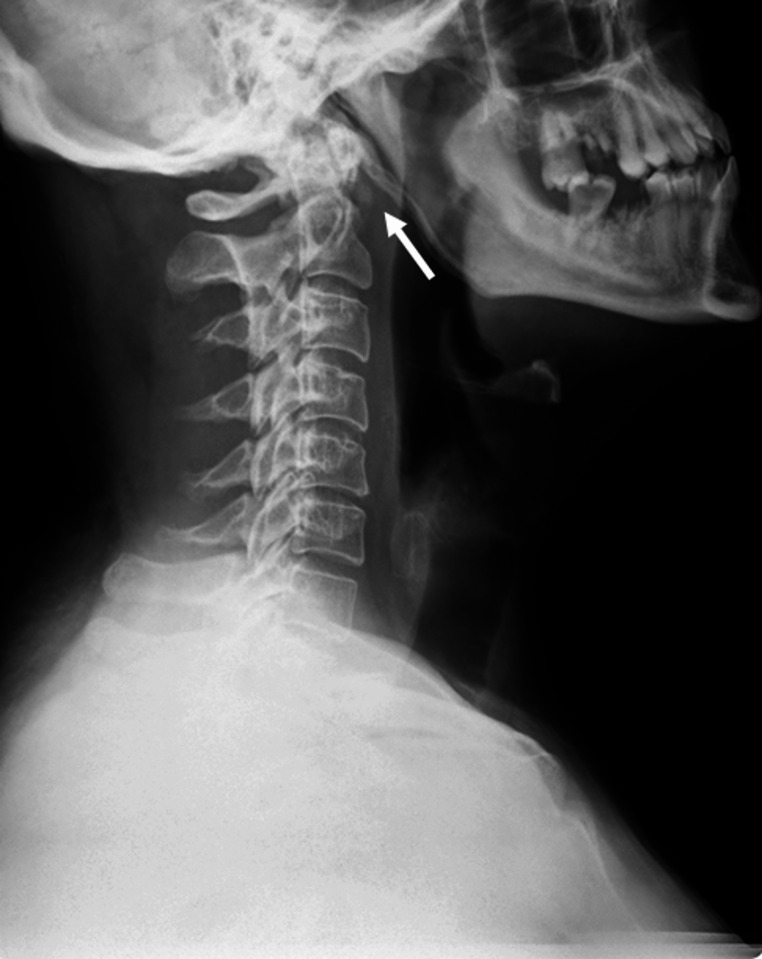
Lateral cervical spine radiography of a patient with *Longus Colli Calcific Tendinitis* where a calcific deposit (white arrow) is clearly identified on the anteroinferior border of the anterior arch of C1

**Fig. 2:**
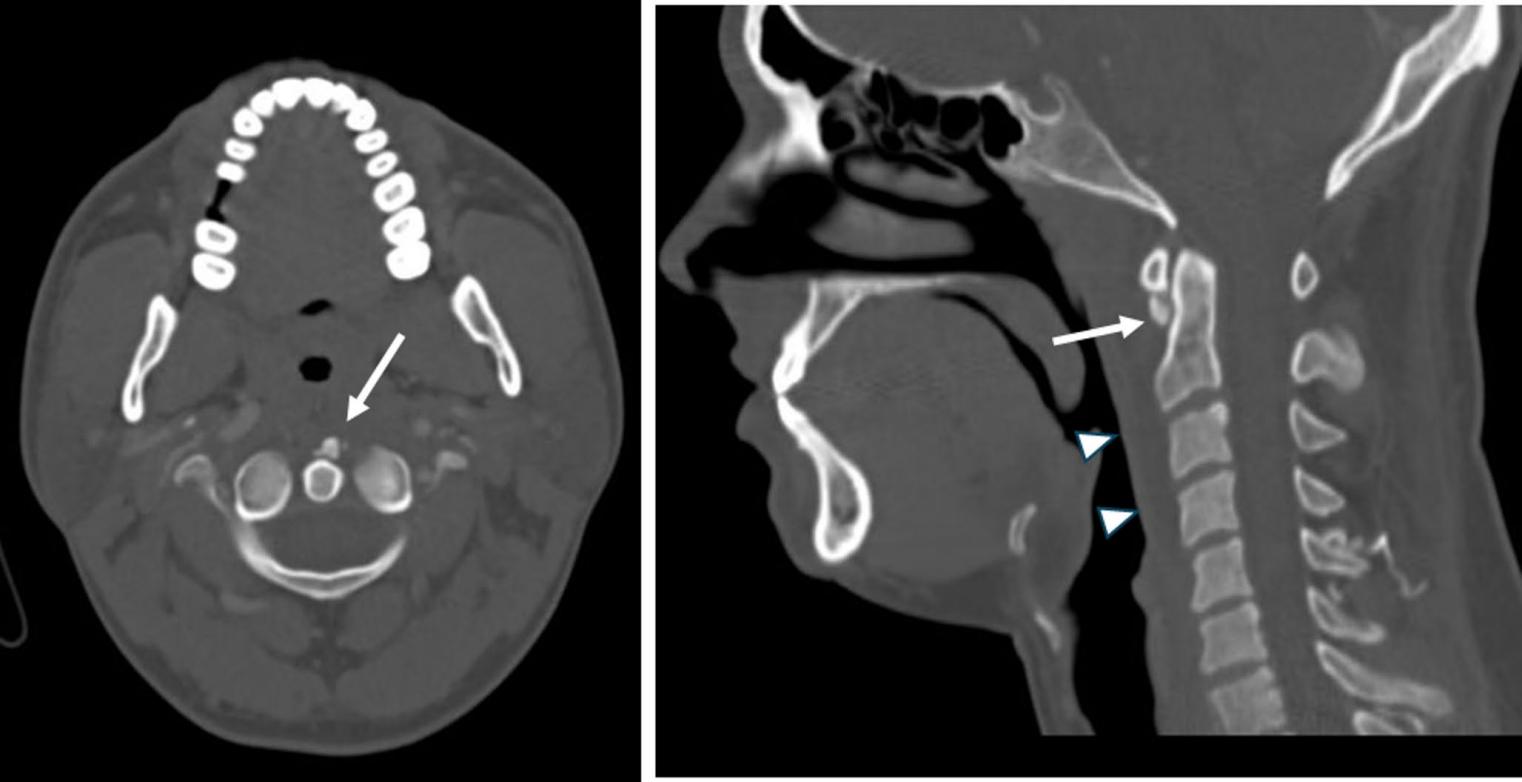
**Left**: Axial CT of a patient with a clear calcification on the anteroinferior border of the anterior arch of C1 (white arrow). **Right**: On Sagittal CT there is also a thin hypodense tissue (white arrow heads) that runs from C1 to C5 corresponding to retropharyngeal edema

**Fig. 3:**
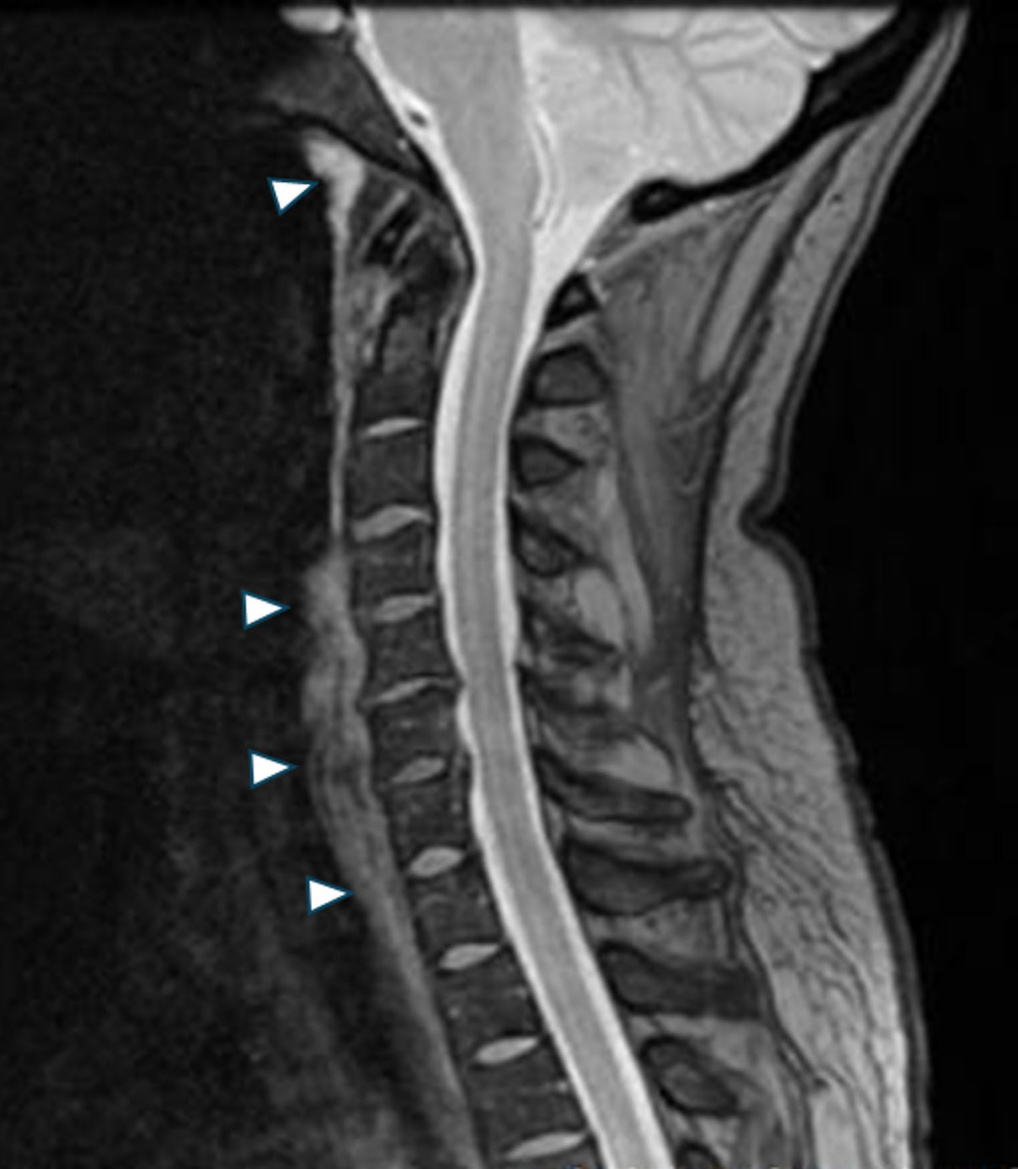
Sagittal T2 MRI of a patient with *Longus Colli Calcific Tendinitis*, it is not possible to clearly identify a calcific deposit on the anteroinferior border of the anterior arch of C1. It is notable the presence of a hyperintense image along the anterior aspect of the cervical spine running from C1 to T3 in the retropharyngeal space, corresponding with soft tissue edema (white arrow heads)

Usual imaging studies consist of X ray, CT and Contrast MRI. Plain lateral radiography is usually unable to show calcific deposits on the anterior C1 arch [Fig. 1]. CT is currently the gold standard for identification of the calcific deposit usually at C1-C2 level on the anteroinferior margin of the anterior arch of C1 [Fig. 2]. It is also useful to evaluate the retropharyngeal space in patients with LCCT where it is often found to be slightly increased (should be less than 4 mm in a normal CT) but without clear signs that would be expected to find in a retropharyngeal abscess like hydro-aerial collection, rim enhancement or suppurative lymphadenopathy. On the other hand, contrast MRI is not very useful to identify the calcific deposits on the C1 arch [Fig. 3], but its usefulness lies in the fact that it can easily show rim enhancement in the case of an abscess and also, it is useful to identify cases of spondylodiscitis [11, 21].

As mentioned before, differential diagnosis such as retropharyngeal abscess, meningitis [22] and spondylodiscitis, is challenging, so the physician must know clearly how to identify the clinical and radiological signs of a LCCT, of special importance is the retropharyngeal abscess since it is the most important mimicker of this pathology, and mistakenly diagnosing them may lead to needless interventions such as biopsy/aspiration, patient discomfort and unnecessary antibiotic prescription [6, 21, 23, 24].

Treatment usually consists of non-steroidal anti-inflammatory drugs (NSAIDs) as the first line of treatment, in severe cases or in patients with intolerance to NSAIDs corticosteroids and opioids may be prescribed [3, 6, 23, 25–27]. Some authors recommend rest and neck immobilization but there is lacking evidence that these interventions are useful.

## Conclusion

LCCT is a self-limited pathology that is caused by a foreign-body type reaction in the retropharyngeal space secondary to the degradation and resorption of calcium hydroxyapatite deposits usually found at the anteroinferior border of the anterior C1 arch. It most commonly present as a triad consisting of neck pain, neck stiffness and odynophagia, but it can also be accompanied by torticollis and fever with a slight elevation of ESR and CRP. The gold standard for diagnosis is Contrast CT, where it is possible to identify C1-C2 calcific deposits and retropharyngeal edema. It is necessary to create awareness of this pathology amongst physicians because it can mimic more serious illness like retropharyngeal abscess, meningitis and spondylodiscitis and this may lead to unnecessary expenditures and antibiotic usage.

## Data Availability

No datasets were generated or analysed during the current study.
